# Supplementary material for: Biopreservation and reversal of oxidative injury during blood storage by a novel curcumin-based gel formulation
Source: Sci Rep. 2024 Dec 28;14:31441. doi: 10.1038/s41598-024-82943-1 (PMC11682427; doi:10.1038/s41598-024-82943-1)

**Supplemental Results**

**Curcuminoid gel reduces the prooxidant ferryl hemoglobin in stored blood.**

We followed the spontaneous oxidation of blood for 42 days at 4°C (normal blood bank storage temperature) by monitoring spectral changes in the heme iron with and without the additive (50 µM curcuminoids). Oxidation of blood under these conditions exhibited little change in the formation of the oxidized Hb (metHb) over the 42-day period (Supplemental Figure 1A) as we have previously reported^1^. No major changes were noted when these mixtures were treated at 4°C. However, higher autoxidation rate and metHb formation are normally doubled during spontaneous oxidation at 37°C as we and others have previously observed ^1,2^.

It is known that RBCs under some pathological or storage conditions are exposed to oxidants, such as peroxide in blood^3,4^, which can accelerate oxidation reactions and oxidative changes in Hb. Supplemental Figure 1A shows spectral data collected during the spontaneous oxidation (autoxidation) of RBCs in air with little or no change in the oxidation rate and metHb formation. Supplemental Figure 1B & C shows spectral data collected during the exposure of 1-day-old (B) and 7-day old RBCs (C) to 20 mM of hydrogen peroxide. During peroxide-mediated oxidation, a higher oxidation Hb intermediate, specifically, the ferryl state (HbFe^4+^), was formed. The HbFe^4+^ spectrum is characterized by major peaks at 541 nm and 578 nm and a flattened region between 600 and 700 nm^5^. The amount of transient HbFe^4+^ formed in the reaction was captured by the addition of sodium sulfide to convert the ferryl species to a spectrally more distinct and stable sulfHb, which has a characteristic maximum optical absorbance band at 620 nm. Supplemental Figure 1B & C depicts spectral changes during the addition of peroxide (20 nm) to Hb (60µM) from 1-day old RBCs stored with 500µM curcuminoids. Sodium sulfide (2mM) was then added to derivatize ferrylHb into sulfhemoglobin. A total of 6.5 µm sulfHb (ferrylHb) formed immediately after the addition of Na_2_S. This was reduced to 2.3 µm sulfHb after addition of the curcumin. This represented a 64% reduction in the ferryl content. We noted a slightly smaller (50%) reduction in the ferryl content in the 7-day old RBC solution after treatment with curcuminoids.


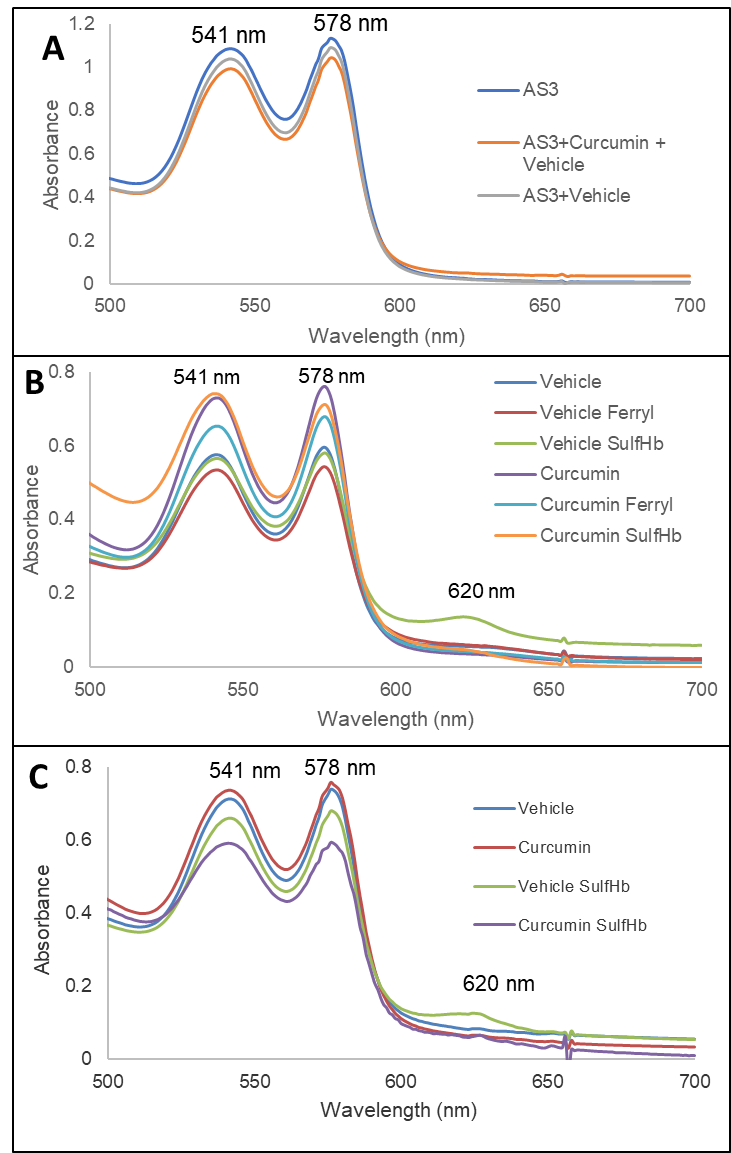


**Supplemental Figure 1: Spontaneous and peroxide-mediated oxidation of RBCs in the presence or absence of curcumin.** (A) Spontaneous oxidation (autoxidation) of hemoglobin (60µM) in AS-3 solutions at room temperature followed for 35 days. Spectral analysis in the visible region of RBC solutions were followed spectrophotometrically in an Agilent Spectrophotometer. Three (3) spectral overlays of RBCs in AS-3 solutions, RBCs + curcumin, and vehicle alone are shown. Multicomponent analysis based on extinction coefficients were used to calculate oxy, met and ferryl Hb levels (B and C) corresponding to hydrogen peroxide-induced oxidation of 1-day and 7-day samples of RBCs, respectively. The samples were hemolyzed, 60 µM of hemoglobin was treated with 20 mM hydrogen peroxide, followed by addition of 2 mM Na_2_S to derivatize the ferryl formed in the reaction. Spectra were collected for vehicle (blue), curcumin (red), vehicle with Na_2_S (gray) and Curcumin with Na_2_S (yellow).

**References:**

1 Jana, S., Kassa, T., Wood, F., Hicks, W. & Alayash, A. I. Changes in hemoglobin oxidation and band 3 during blood storage impact oxygen sensing and mitochondrial bioenergetic pathways in the human pulmonary arterial endothelial cell model. *Front Physiol* **14**, 1278763 (2023). <https://doi.org:10.3389/fphys.2023.1278763>

2 Roch, A. *et al.* Transition to 37°C reveals importance of NADPH in mitigating oxidative stress in stored RBCs. *JCI Insight* **4** (2019). <https://doi.org:10.1172/jci.insight.126376>

3 Forman, H. J., Bernardo, A. & Davies, K. J. A. What is the concentration of hydrogen peroxide in blood and plasma? *Archives of Biochemistry and Biophysics* **603**, 48-53 (2016). <https://doi.org:https://doi.org/10.1016/j.abb.2016.05.005>

4 Alayash, A. I. Hemoglobin Oxidation Reactions in Stored Blood. *Antioxidants* **11**, 747 (2022).

5 Jia, Y., Buehler, P. W., Boykins, R. A., Venable, R. M. & Alayash, A. I. Structural basis of peroxide-mediated changes in human hemoglobin: a novel oxidative pathway. *J Biol Chem* **282**, 4894-4907 (2007). <https://doi.org:10.1074/jbc.M609955200>

**Uncropped gel/blot images**


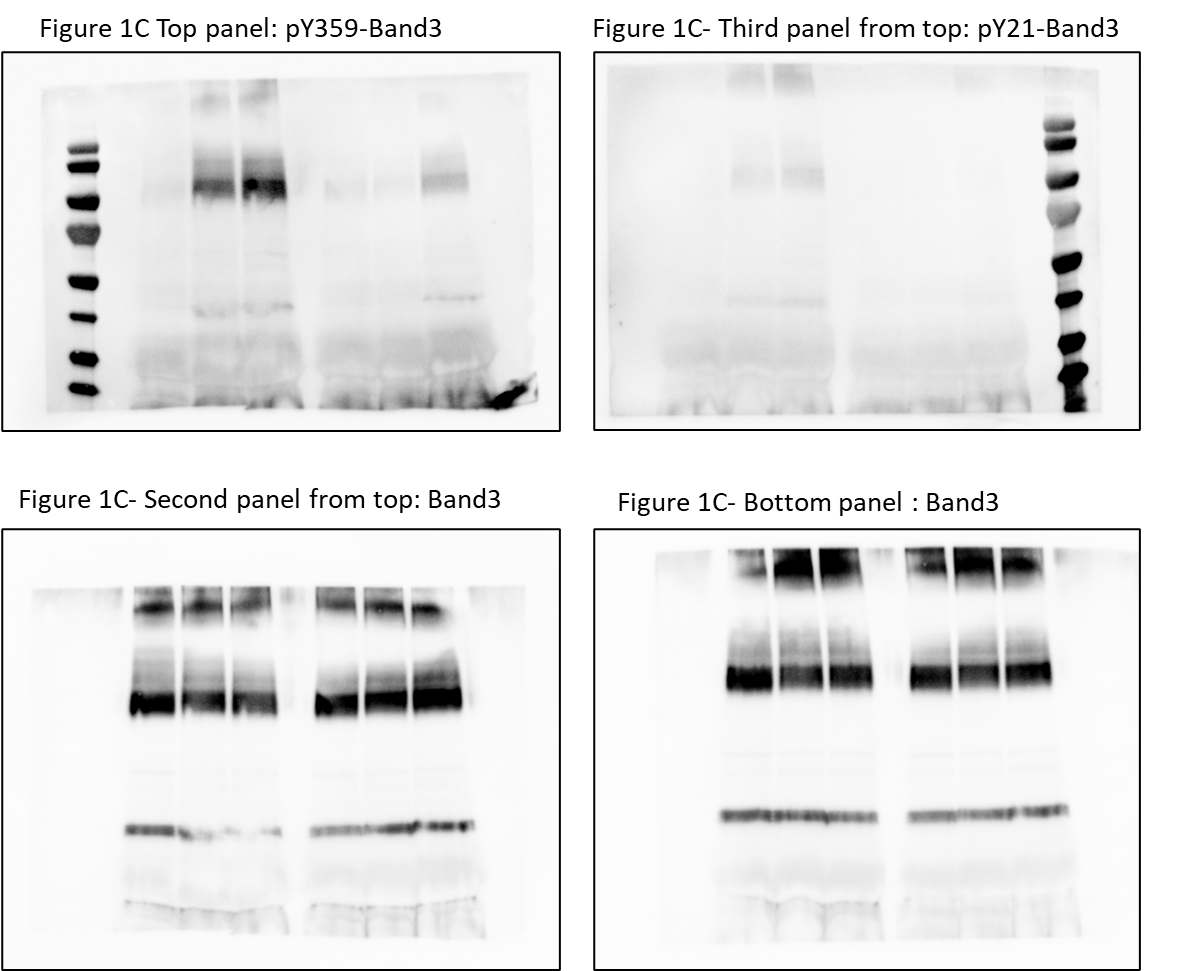


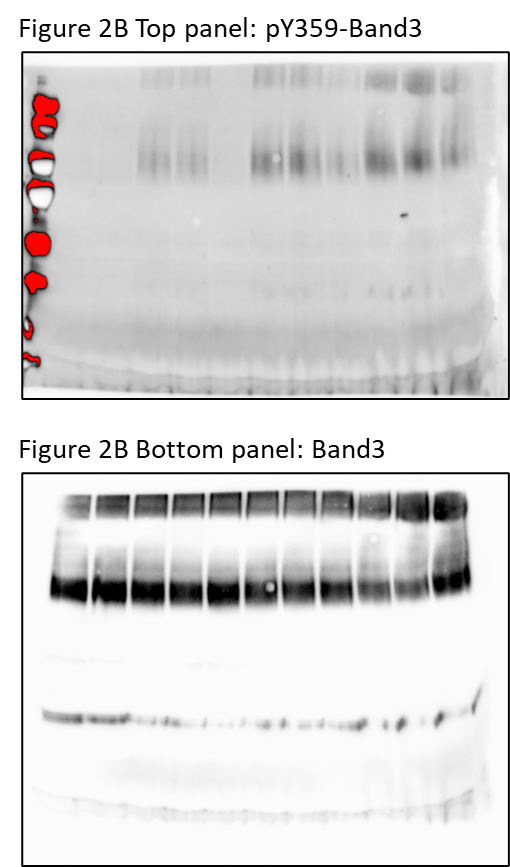

Supplement: Supplementary file 1 — Supplementary Material 1 [file 41598_2024_82943_MOESM1_ESM.docx]
